# Pulmonary hypertension evaluation by Doppler echocardiogram in children and adolescents with mouth breathing syndrome^☆^^☆☆^

**DOI:** 10.1016/j.bjorl.2016.03.020

**Published:** 2016-06-08

**Authors:** Marcela Silva Lima, Carolina Maria Fontes Ferreira Nader, Letícia Paiva Franco, Zilda Maria Alves Meira, Flavio Diniz Capanema, Roberto Eustáquio Santos Guimarães, Helena Maria Gonçalves Becker

**Affiliations:** a Universidade Federal de Minas Gerais (UFMG), Departamento de Cirurgia e Oftalmologia, Belo Horizonte, MG, Brazil; b Faculdade de Saúde e Ecologia Humana (FASEH), Vespasiano, MG, Brazil; c Faculdade de Saúde e Ecologia Humana (FASEH), Fundação de Amparo a Pesquisa do Estado de Minas Gerais (FAPEMIG), Programa Institucional de Bolsas de Iniciação Científica e Tecnológica (PROBIC), Vespasiano, MG, Brazil; d Universidade Federal de Minas Gerais (HC-UFMG), Hospital das Clínicas, Belo Horizonte, MG, Brazil; e Universidade Federal de Minas Gerais (UFMG), Faculdade de Medicina, Belo Horizonte, MG, Brazil; f Universidade Federal de Minas Gerais (UFMG), Departamento de Pediatria, Belo Horizonte, MG, Brazil; g Núcleo de Inovação Tecnológica, Fundação Hospitalar do Estado de Minas Gerais (FHEMIG), Santa Efigênia, MG, Brazil; h Universidade Federal de Minas Gerais (UFMG), Faculdade de Medicina, Departamento de Oftalmologia, Otorrinolaringologia e Fonoaudiologia, Belo Horizonte, MG, Brazil; i Universidade Federal de Minas Gerais (HC-UFMG), Hospital das Clínicas, Centro Multidisciplinar de Atenção ao Respirador Oral, Belo Horizonte, MG, Brazil

**Keywords:** Mouth breathing, Adenoidectomy, Pulmonary hypertension, Doppler echocardiography, Rhinitis, Respiração bucal, Adenoidectomia, Hipertensão pulmonar, Ecodopplercardiografia, Rinite

## Abstract

**Introduction:**

Adenotonsillar hyperplasia (ATH) and allergic rhinitis (AR) are the most common causes of upper airway obstruction in children. Such diseases, by affecting the upper airways, can cause chronic alveolar hypoventilation, pulmonary vasoconstriction and pulmonary hypertension, which in some cases, are irreversible.

**Objective:**

This cross-sectional study aimed to evaluate the prevalence of pulmonary hypertension in two groups of mouth-breathing (MB) 2–12 years old children with ATH and isolated allergic rhinitis, through Doppler echocardiography.

**Methods:**

54 patients with ATH and indications for adenoidectomy and/or tonsillectomy and 24 patients with persistent allergic rhinitis were selected and submitted to Doppler echocardiography. The Systolic Pulmonary Artery Pressure (SPAP) was determined by tricuspid regurgitation and the Mean Pulmonary Artery Pressure (MPAP) was calculated from the SPAP. Similar measurements were carried out in 25 nasal breathing (NB) individuals.

**Results:**

The mean MPAP and SPAP were higher in the MB than in the NB group (17.62 ± 2.06 [ATH] and 17.45 ± 1.25 [AR] vs. 15.20 ± 2.36 [NB] mmHg, *p* < 0.005, and 25.61 ± 3.38 [ATH] and 25.33 ± 2.06 [AR] vs. 21.64 ± 3.87 [NB] mmHg, *p* < 0.005, respectively) and the mean acceleration time of pulmonary flow trace (Act) was higher in the NB than in the MB group (127.24 ± 12.81 [RN] vs. 114.06 ± 10.63 ms [ATH] and 117.96 ± 10.28 [AR] MS [AR]; *p* < 0.0001).

**Conclusion:**

None of the MB children (ATH and AR) met the PH criteria, although individuals with both ATH and isolated AR showed significant evidence of increased pulmonary artery pressure by Doppler echocardiography in relation to NB individuals. No differences were observed between the ATH and AR groups.

## Resumo

**Introdução:**

A hiperplasia adenotonsilar (HAT) e a rinite alérgica (RA) consistem nas causas mais comuns de obstrução de vias aéreas superiores em crianças. Tais afecções, ao comprometer a via aérea superior, podem ocasionar hipoventilação alveolar crônica, vasoconstrição pulmonar e hipertensão pulmonar, em alguns casos irreversível.

**Objetivo:**

Este estudo transversal objetivou avaliar a prevalência de hipertensão arterial pulmonar em dois grupos de crianças respiradoras orais (RO): com HAT e rinite alérgica isolada, de 2 a 12 anos, por meio de exame ecodopplercardiográfico.

**Método:**

Foram selecionados e submetidos à ecodopplercardiografia 54 pacientes com HAT com indicação de adenoidectomia e/ou tonsilectomia e 24 pacientes com rinite alérgica persistente. A pressão sistólica da artéria pulmonar (PSAP) foi determinada pela regurgitação tricúspide e a pressão média da artéria pulmonar (PMAP) foi calculada a partir da PSAP. Determinações similares foram realizadas em 25 respiradores nasais (RN).

**Resultados:**

As médias da PMAP e da PSAP foram maiores nos grupos de RO do que nos RN (17,62 ± 2,06 [HAT] e 17,45 ± 1,25 [RA] vs. 15,20 ± 2,36 [RN] mmHg; *p* < 0,005; e 25,61 ± 3,38 [HAT] e 25,33 ± 2,06 [RA] vs. 21,64 ± 3,87 [RN] mmHg; *p* < 0,005; respectivamente) e a média do tempo de aceleração do traçado do fluxo pulmonar (TAc) foi maior nos RN que nos grupos de RO (127,24 ± 12,81 [RN] vs. 114,06 ± 10,63 ms [HAT] e 117,96 ± 10,28 [RA] MS [RA]; *p* < 0,0001).

**Conclusão:**

Nenhuma criança respiradora oral (HAT e RA) preencheu os critérios de HP, embora tanto os portadores de HAT quanto de RA isolada apresentaram evidências significativas de aumento da pressão arterial pulmonar pela ecodopplercardiografia em relação aos respiradores nasais. Não se observou diferença entre os grupos HAT e RA.

## Introduction

Mouth breathing syndrome (MBS) refers to the clinical condition in which the individual has a breathing pattern performed predominantly through the oral cavity for a period longer than six months.^1^, ^2^ Among the etiologies, allergic rhinitis is the most common cause of chronic upper airway obstruction (UAO),^3^ accounting for up to 85% of cases. The hypertrophy of adenoids and/or tonsils corresponds to 79.2%, being the main cause of Obstructive Sleep Apnea (OSA) in children.^1^, ^4^, ^5^, ^6^, ^7^, ^8^, ^9^

Chronic nasal obstruction and the consequent OBS result in an insufficient supply of oxygen and alveolar ventilation during the night, causing hypoventilation, hypoxemia and hypercapnia that can lead to OSA and pulmonary vasoconstriction, which, if persistent, results in pulmonary hypertension (PH).^6^, ^7^, ^8^, ^9^, ^10^, ^11^, ^12^, ^13^, ^14^ PH in children is defined as Mean Pulmonary Artery Pressure (MPAP) ≥25 mmHg or Systolic Pulmonary Artery Pressure (SPAP) ≥35 mmHg at rest.^6^, ^15^ Although there are few reports of severe manifestations such as cor pulmonale and death,^16^, ^17^, ^18^, ^19^, ^20^, ^21^ PH in its initial phase has been very prevalent in some studies involving up to 84% of mouth breathers (MB).^11^ Nevertheless, underdiagnosis is observed in the initial phase of PH due to the scarcity of cardiovascular symptoms.^11^ An early approach, with adenotonsillectomy and treatment of allergic rhinitis, promptly reverses elevated pressures in the pulmonary artery.^6^, ^7^, ^8^, ^9^, ^12^, ^13^, ^14^, ^22^

The Doppler echocardiographic evaluation is a non-invasive method that allows the estimation of the MPAP and SPAP values with a sensitivity of 79–100% and a specificity of 68–98%.^15^, ^23^ In spite of its low-cost and potential for an early assessment of cardiac complications, it is not routinely performed in patients with UAO, being restricted to severe cases of OSA with evident cardiovascular deterioration.^6^, ^7^, ^9^, ^12^, ^13^

This study aimed to evaluate the presence of PH in MB with UAO caused by ATH and AR in children and adolescents treated in a referral center through the Doppler echocardiographic method.

## Methods

This study was approved by the Research Ethics Committee (CAAE number: 20931213.0.0000.5149). The patients were informed about the study and its objectives and were asked to sign the free and informed consent form, after assisted reading and guidance.

The sample consisted of children and adolescents aged two to twelve years, MB with ATH and AR, treated as first consultation at the Mouth Breather Outpatient Clinic, in addition to patients with no MB complaints, treated at the pediatric and speech therapy outpatient clinic for other complaints that were allocated in the nasal breather (NB) group.

Patient sample size was calculated using two comparative studies^12^, ^13^ involving two proportions: the comparison of pulmonary hypertension (PH) prevalence in the MB versus the prevalence of PH in the NB group. Considering the prevalence of 10%^12^ in NB and 60%^13^ or more in MB, in order to detect differences between the two groups in at least 50% (*α* = 0.05 and *β* = 0.10), it would be necessary to have at least 21 patients in each group.

At the selection procedure, patients underwent anamnesis, complete otorhinolaryngological examination, allergologic evaluation with skin test puncture and a multidisciplinary evaluation (physical therapist, orthodontist and speech therapist). Together with the subjective evaluation of nasal obstruction, all patients underwent anterior active rhinomanometry (RAA) and Doppler echocardiographic assessment. Patients who met the following criteria were selected:

**Inclusion criteria** – We included MB patients with tonsillar hyperplasia grade III or IV and/or adenoid hyperplasia with nasopharynx obstruction ≥75%,^24^ confirmed by otorhinolaryngological examination and fibronasopharyngolaryngoscopy (ATH group) and clinical diagnosis of rhinitis with positive skin test (AR group).

• ATH group: mouth breathing children with hyperplasia of the palatine tonsil grade III and grade IV and/or adenoid hyperplasia with nasopharynx obstruction ≥75%, confirmed by otorhinolaryngological examination and fibronasopharyngolaryngoscopy, with or without positive skin test;
• AR group: mouth breathing children without obstructive adenotonsillar hyperplasia and with nasal hyperreactivity and positive skin test;
• NB group: nasal breathing children without adenotonsillar hyperplasia and negative skin test.

**Exclusion criteria** – Patients with heart disease; previous craniofacial and respiratory system surgeries; skin lesions that prevented the performance of skin allergic test (SAT); respiratory infection in the upper and low respiratory tracts in the last 14 days; chronic comorbidities; to be using or having used the following medications: intranasal corticosteroids, anticholinergics, systemic or topical vasoconstrictor, nasal cromolyn or other nasal decongestant in the past four weeks; antihistamines in the last two weeks; oral corticosteroids and leukotriene receptor antagonist in the last eight weeks; specific immunotherapy in the past 3 months; and those who did not sign the free and informed consent (FIC) form were excluded.^4^, ^9^, ^12^, ^25^, ^26^, ^27^

### Operational definitions

The study consisted of three stages: 1 – selection of MB and NB in pediatric clinics and referral to the specialized center; 2 – evaluation of referrals for diagnostic confirmation or exclusion of the OBS and for etiological diagnosis and classification of ATH; 3 – performing the Doppler echocardiography to diagnose PH.

All patients, MB and NB, were submitted to complete otorhinolaryngological examination, allergy skin test, fibronasopharyngolaryngoscopy and active anterior rhinometry. Tonsil size was classified according to the Brodsky's criteria,^27^ considering the degree of oropharyngeal obstruction. Adenoid hyperplasia was rated by nasal endoscopy according to the percentage of choanal lumen obstruction degree.^1^, ^24^

All patients underwent Doppler echocardiography with color flow mapping using Phillips^®^ IE 33 device. For the SPAP analysis, tricuspid regurgitation measurement was performed using the apical window in the apical four-chamber view, measuring systolic peak velocity and using Bernoulli's formula; the MPAP estimate was performed using the formula: MPAP = 0.61 × SPAP + 2 mmHg^28^; in addition to the calculation of the time of acceleration of the pulmonary flow (TAc), by measuring the interval between the beginning and the peak of the pulmonary flow wave. All measurements were performed by calculating the average of three heartbeats and the examinations were carried out by two echocardiographers experienced in the method, without knowledge of the patients’ clinical data. The upper limit of MPAP was considered as 25 mmHg and SPAP as 35 mmHg.^6^, ^15^ The echocardiographers were unaware of which group the patient belonged to.

### Analyzed variable

#### MPAP and SPAP

According to the recommendations of the “Guidelines for the diagnosis and treatment of pulmonary hypertension”,^15^ PH consists in MPAP ≥ 25 mmHg. When MPAP is between 20 mmHg and 24 mmHg, an altered MPAP is considered, but as we do not know the significance of its clinical impact, it is not considered as PH. The value for SPAP ≥ 35 mmHg is also considered diagnostic in some studies, as well as TAc ≤ 100 ms.^12^, ^28^

### Statistical analysis

Categorical data are shown as numbers and percentages, and continuous data as mean and standard deviation. Categorical variables of patients and controls were compared using the chi-square test. Continuous variables were analyzed by Student's *t* test. Comparisons between the study groups were carried out through bilateral hypothesis tests considering a significance level of 5% (=0.05).

## Results

The study population consisted of 54 chronic mouth breathers with ATH and surgical indication for adenoidectomy and/or tonsillectomy (31 males, with a mean age of 6.04 ± 2.24 years and mean BMI of 16.57 ± 2.63) and 24 patients with persistent AR (14 males, with a mean age of 7.42 ± 2.05 years and mean BMI of 16.08 ± 2.18) compared to 25 healthy patients without UAO. There was a statistically significant difference in age and height, with the mean height and age being lower in the ATH group. However, there were no significant differences in gender, weight and body mass index (BMI) (Table 1).

**Table 1:** Demographic and anthropometric data.

	NB (*n* = 25)		ATH (*n* = 54)		AR (*n* = 24)	
	Mean	(SD)	Mean	(SD)	Mean	(SD)
Male gender (%/*n*)	32.0%	8	57.4%	31	58.3%	14
Age (years)^a^	7.30	(2.47)	6.04^b^	(2.24)	7.42	(2.05)
Weight (kg)	27.92	(11.07)	22.83	(8.20)	25.36	(8.79)
Height (m)^a^	1.27	(0.17)	1.15^b^	(0.15)	1.23	(0.15)
BMI (kg/m^2^)	16.24	(2.77)	16.57	(2.63)	16.08	(2.18)

a Statistically significant difference between group with AR and group with ATH (*p* < 0.05).

b Statistically different from the NB group (*p* < 0.05).

Regarding the history obtained at the referral outpatient clinic and directed to parents and/or guardians of the MB about the behavior of their children during sleep, all of them reported mouth breathing. Among the complaints related to OSA, all patients with adenotonsillar hypertrophy and 19 patients (79.2%) from the rhinitis group complained of snoring, while breathing pauses suggestive of apnea were prevalent in 62.7% and 39.1% in the ATH and AR groups, respectively. Regarding adenoid hyperplasia with indication for adenoidectomy, 42 patients (77.7%) had adenoids occupying 75% or more of the nasopharynx, and 17 (31.4%) had adenoids occupying 90% or more. Allergic rhinitis with a positive skin test was detected in 18 (35.3%) patients with ATH (Table 2).

**Table 2:** Data obtained from the history and physical examination of patients at the first consultation at the referral clinic.

	NB (*n* = 25)		ATH (*n* = 54)		AR (*n* = 24)	
	%	*n*	%	*n*	%	*n*
Asthma^a^	4.0%	1	19.6%^b^	10	50.0%^b^	12
+Skin test^a^	0.0%	0	35.3%^b^	18	100.0%^b^	24
Snoring	0.0%	0	96.2%^b^	51	79.2%^b^	19
Apnea	0.0%	0	62.7%^b^	32	39.1%^b^	9
Bruxism	28.0%	7	42.0%	21	50.0%	12
Headache	28.0%	7	38.9%	21	29.2%	7
% R Adenoid (mean/SD)^a^	11.8%	(15.1%)	76.7%^b^	(19.2%)	19.2%	(14.8%)
% L Adenoid (mean/SD)^a^	12.2%	(15.4%)	76.4%^b^	(17.9%)	19.0%	(14.7%)
R tonsillar grade^a^	I: 18; II: 7; III: 0; IV: 0		I: 7; II: 11; III: 24; IV: 12^b^		I: 13; II: 10; III: 1; IV: 0	
L tonsillar grade^a^	I: 17; II: 8; III: 0; IV: 0		I: 7; II: 8; III: 24, IV: 15^b^		I: 15; II: 9; III: 0; IV: 0	

a Statistically significant difference between group with AR and group with ATH (*p* < 0.05).

b Statistically different from the NB group (*p* < 0.05). Tonsillar grade – I < 25% II ≥ 25% and <50% III ≥ 50% and <75%, IR ≥ 75%.

Among the echocardiographic findings, we observed a difference regarding MPAP and SPAP, being higher in the MB than the NB group (17.62 ± 2.06 (ATH) and 17.45 ± 1.25 (AR) vs. 15.20 ± 2.36 and 25.61 ± 3.38 mm Hg (ATH) and 25.33 ± 2.06 (AR) vs. 21.64 ± 3.87 mmHg, *p* < 0.005, respectively), while TAc was higher in NB (127.4 ± 12.81 vs. 114.06 ± 10.63 ms (ATH) and 117.96 ± 10.28, *p* < 0.0001). There was no significant difference between SPAP, MPAP and TAc values among mouth breathers when considering the different etiologies, ATH and AR (Table 3).

**Table 3:** Mean values of SPAP, MPAP and TAc.

	NB (*n* = 25)	ATH (*n* = 54)	AR (*n* = 24)
	Mean (SD)	Mean (SD)	Mean (SD)
SPAP (mmHg)	21.64 (3.87)	25.61^a^ (3.38)	25.33^a^ (2.06)
MPAP (mmHg)	15.20 (2.36)	17.62^a^ (2.06)	17.45^a^ (1.25)
TAc (mmHg)	127.24 (12.81)	114.06^a^ (10.63)	117.96^a^ (10.28)

a Statistically different from the NB group (*p* < 0.05). Statistically significant difference between the AR group and group with ATH (*p* < 0.05).

**Figure 1:**
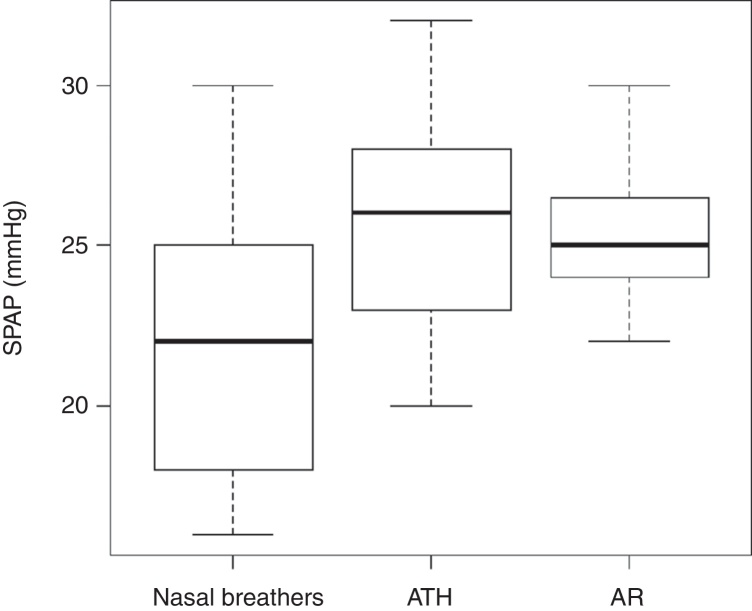
Box plot graph showing the association between the SPAP in the MB and NB groups.

No child had PH at the echocardiography. Seven patients with ATH (12%) had MPAP ≥ 20 mmHg, but none had MPAP ≥ 25 mmHg. The rhinitis group and NB group had a patient with MPAP ≥ 20 mmHg each. TAc ranged from 84 to 136 ms in the ATH group, from 96 to 142 ms in patients with AR and 100 to 146 ms in the group of nasal breathers. TAc ≤ 100 ms was found in six patients with adenotonsillar hypertrophy (11%), in one patient of the NB group and in one patient of the AR group. SPAP ranged from 19 to 32 mmHg in patients from the ATH group and from 22 to 32 in the AR group, whereas in the NB group SPAP ranged from 16 to 30 mmHg (Fig. 1).

## Discussion

In this study, statistically significant differences were observed between the oral and nasal breathers in relation to PH measures (SPAP, MPAP and TAc), which indicate the risk of development of future irreversible cardiopulmonary complications. However, no patient had a diagnosis of PH, contrary to what is described in the literature.

Naiboglu et al. assessed the change in Pulmonary Artery Pressure (PAP) in children with adenotonsillectomy indication compared to a control group. Of the 39 patients with upper airway obstruction, 84% were considered hypertensive due to increased MPAP, whereas in the control group only two patients were classified as hypertensive. However, the used cutoff value was 20 mmHg and the calculation of MPAP was performed through TAc, which is an unreliable parameter when it is not corrected by the heart rate.^12^

In a study carried out by Yilmaz^13^ et al. (2005), 52 children were randomly selected, aged between 4 and 11 years, with the diagnosis of UAO secondary to ATH and 33 children in the control group. MPAP levels were compared between cases and controls, and the first group had significantly higher blood pressure levels (MPAP of 23.13 ± 7.68 in cases and 16.11 ± 7.24 in controls).

As in the Naiboglu work, Yilmaz et al. classified as hypertensive those patients with MPAP > 20 mmHg. However, the prevalence of PH was lower with the involvement of 51% of patients with adenotonsillectomy indication, compared to 3% in the control group. Both studies evaluated the reversal of MPAP values after the adenotonsillectomy and found similar pressure values to those in the control group after the procedure.^12^, ^13^

In addition to the aforementioned studies, a recent one by Martha et al. analyzed SPAP and MPAP values in 33 children with adenotonsillar hypertrophy and compared them with 10 control patients. The diagnosis of PAH was achieved through Doppler echocardiographic evaluation and patients with SPAP ≥ 30 mmHg or MPAP ≥ 25 mmHg were considered hypertensive. The prevalence of hypertension was 36% in the ATH group, a lower value than in other studies. The evaluation of systolic pressure was made by tricuspid regurgitation, whereas in patients without this regurgitation, MPAP was calculated from TAc. In the group of 19 patients in which the evaluation was performed by the Mahan formula, a calculation that depends on the TAc value, the prevalence of PH was higher.^6^

In contrast to the aforementioned studies, the present study also assessed the impact of allergic rhinitis on pulmonary pressure and compared the findings to the group with adenotonsillar hypertrophy. Although there have been few studies on AR and PH,^9^, ^10^ we can demonstrate that the mean pulmonary pressure is high in rhinitis and comparable to those in patients with significant adenotonsillar hypertrophy.

The association between allergic rhinitis (AR) and pulmonary hypertension was also evaluated in a study with 35 patients that correlated symptom severity with changes in echocardiography in patients with seasonal allergic rhinitis. The literature showed an increase in PAP during patients’ symptomatic periods, with a reduction in PAP values during the asymptomatic period. The symptom that showed a higher correlation with PAP results was nasal obstruction severity, indicating a previously discussed association of nasal resistance increase with cardiopulmonary alterations.^10^

Some^12^ studies use TAc to evaluate PH with the reference value (limit of normality) of 100 ms. This method is often used in adults, but has limitations in children with elevated heart rate, as it can significantly influence the pattern of the outflow tract velocity curve and the acceleration time. Therefore, other methods are recommended, particularly those using continuous Doppler, since it allows indirect measurement of the Systolic Pulmonary Artery Pressure by assessing tricuspid regurgitation.^29^ In the present study, we used the measure derived from tricuspid regurgitation, the most recommended method and the one with lower inter and intraobserver variability.

This study is relevant for the literature, as it can influence clinical practice through a new approach proposal for MB, in addition to warn against cardiopulmonary risks that are not as yet well established. The care of patients with UAO should not focus only on patients with ATH, as changes in pulmonary pressure were comparable in patients with ATH and AR. Thus, the early identification of patients at risk for PH is important, as it is directly related to prognosis because of the potential reversibility of the picture, when corrective surgical procedure and/or drug treatment indication are carried out in a timely manner.^6^, ^9^, ^12^, ^13^

## Conclusion

Although no MB in the assessed sample was diagnosed with PH, both SPAP and MPAP, measured by Doppler echocardiography, showed increased means when compared to nasal breathers, i.e., the normal standard. This fact may pose a greater risk for future heart complications in patients with MBS.

Patients with allergic rhinitis showed elevated values of MPAP and SPAP that were similar to those obtained in patients with upper airway obstruction due to adenotonsillar hypertrophy.

## Conflicts of interest

The authors declare no conflicts of interest.

## References

[1] Becker H.M.G., Guimaraes R.E.S., Pinto J.A., Vasconcellos M.C., Leão E. (2012). Pediatria ambulatorial.

[2] Saffer M. (2002). II IAPO/IFOS pediatric ent manual.

[3] Chedid K.A.K., Difrancesco R.C., Junqueira P.A.S. (2004). A influência da respiração oral no processo de aprendizagem da leitura e escrita em crianças pré-escolares. Rev Psicopedag.

[4] Barros Juliana R.C., Becker H.M.G., Pinto J.A. (2006). Avaliação de atopia em crianças respiradoras bucais atendidas em centro de referência. J Pediatr.

[5] Abreu R.R., Rocha R.L., Lamounier J.A., Guerra A.F.M. (2008). Etiologia, manifestações clínicas e alterações presentes nas crianças respiradoras orais. J Pediatr.

[6] Martha A.S., Velho F.J., Eick R.G., Goncalves S.C. (2013). Reversal of pulmonary hypertension in children after adenoidectomy or adenotonsillectomy. Int J Pediatr Otorhinolaryngol.

[7] Koc S., Aytekin M., Kalay N., Ozcetin M., Burucu T., Ozbek K. (2012). The effect of adenotonsillectomy on right ventricle function and pulmonary artery pressure in children with adenotonsillar hypertrophy. Int J Pediatr Otorhinolaryngol.

[8] Miman M.C., Kirazli T., Ozyurek R. (2000). Doppler echocardiography in adenotonsillar hypertrophy. Int J Pediatr Otorhinolaryngol.

[9] Yüksel H., Coşkun S., Onağ A. (2001). Doppler echocardiographic evaluation of pulmonary arterial pressure in children with allergic rhinitis. Int J Pediatr Otorhinolaryngol.

[10] Bayrak P., Kirmaz C., Sekuri C., Yuksel H. (2007). Is pulmonary arterial pressure affected by allergic rhinitis with nasal obstruction?. Asian Pac J Allergy.

[11] Di Francesco R.C., Fortes F.S.G., Komatsu C.L. (2004). Melhora da qualidade de vida em crianças após adenoamigdalectomia. Rev Bras Otorrinolaringol.

[12] Naiboglu B., Deveci S., Duman D., Kaya K.S., Toros S., Kinis V. (2008). Effect of upper airway obstruction on pulmonary arterial pressure in children. Int J Pediatr Otorhinolaryngol.

[13] Yilmaz M.D., Onrat E., Altuntas A., Kaya D., Kahveci O.K., Ozel O. (2005). The effects of tonsillectomy and adenoidectomy on pulmonary arterial pressure in children. Am J Otolaryngol.

[14] Abdel-Aziz M. (2011). Asymptomatic cardiopulmonary changes caused by adenoid hypertrophy. J Craniofac Surg.

[15] Galie N., Hoeper M.M., Torbicki A., Vachiery J.L., Barbera J.A., Beghetti M. (2009). Guidelines for the diagnosis and treatment of pulmonary hypertension. Eur Heart J.

[16] Spektor S., Bautista A.G. (1956). Respiratory obstruction caused by acute tonsillitis and adenoiditis. J Med.

[17] Noonan J.A. (1965). Reversible cor pulmonale due to hypertrophied tonsils and adenoids: studies in two cases. Circulation.

[18] Menasche V.D., Farrehi C., Miller M. (1965). Hypoventilation and cor pulmonale due to chronic upper airway obstruction. J Pediatr.

[19] Luke M.J., Mehrizi A., Folger G.M., Rowe R.D. (1966). Chronic nasopharyngeal obstruction as a cause of cardiomegaly, cor pulmonale and pulmonary edema. Pediatrics.

[20] Macartney F.J., Panday J., Scott O. (1969). Cor pulmonale as a result of chronic nasopharyngeal obstruction due to hypertrophied tonsils and adenoids. Arch Dis Child.

[21] Cronje R.E., Human G.P., Simson L. (1966). Hypoxemic pulmonary hypertension in children. S Afr Med J.

[22] Schiffmann R., Faber J., Eidelman A.I. (1985). Obstructive hypertrophic adenoids and tonsils as a cause of infantile failure to thrive: reversed by tonsillectomy and adenoidectomy. Int J Pediatr Otorhinolaryngol.

[23] Trow T.K., Mcardle J.R. (2007). Diagnosis of pulmonary arterial hypertension. Clin Chest Med.

[24] Cassano P., Gelardi M., Cassano M., Fiorella M.L., Fiorella R. (2003). Adenoid tissue rhinopharyngeal obstruction grading based on fiberendoscopic findings: a novel approach to therapeutic management. Int J Pediatr Otorhinolaryngol.

[25] EAACI (1993). Position paper: allergen standardization and skin tests. The European Academy of Allergology and Clinical Immunology. Allergy.

[26] McGoon M.D. (2001). The assessment of pulmonary hypertension. Clin Chest Med.

[27] Brodsky L. (1989). Modern assessment of tonsils and adenoids. Pediatr Clin North Am.

[28] Chemla D., Castelain V., Humbert M., Hébert J.L., Simonneau G., Lecarpentier Y. (2004). New formula for predicting mean pulmonary artery pressure using systolic pulmonary artery pressure. Chest.

[29] Rivera I.R., Moisés V.A. (2002). Estimativa da pressão arterial pulmonar pela ecocardiografia nas cardiopatias congênitas com hiperfluxo pulmonar. Rev Bras Ecocardiogr.

